# Seabird Nutrient Subsidies Benefit Non-Nitrogen Fixing Trees and Alter Species Composition in South American Coastal Dry Forests

**DOI:** 10.1371/journal.pone.0086381

**Published:** 2014-01-22

**Authors:** Gilles Havik, Alessandro Catenazzi, Milena Holmgren

**Affiliations:** 1 Resource Ecology Group, Wageningen University, Wageningen, The Netherlands; 2 Department of Zoology, Southern Illinois University Carbondale, Carbondale, Illinois, United States of America; University of California, Berkeley, United States of America

## Abstract

Marine-derived nutrients can increase primary productivity and change species composition of terrestrial plant communities in coastal and riverine ecosystems. We hypothesized that sea nutrient subsidies have a positive effect on nitrogen assimilation and seedling survival of non-nitrogen fixing species, increasing the relative abundance of non-nitrogen fixing species close to seashore. Moreover, we proposed that herbivores can alter the effects of nutrient supplementation by preferentially feeding on high nutrient plants. We studied the effects of nutrient fertilization by seabird guano on tree recruitment and how these effects can be modulated by herbivorous lizards in the coastal dry forests of northwestern Peru. We combined field studies, experiments and stable isotope analysis to study the response of the two most common tree species in these forests, the nitrogen-fixing *Prosopis pallida* and the non-nitrogen-fixing *Capparis scabrida.* We did not find differences in herbivore pressure along the sea-inland gradient. We found that the non-nitrogen fixing *C. scabrida* assimilates marine-derived nitrogen and is more abundant than *P. pallida* closer to guano-rich soil. We conclude that the input of marine-derived nitrogen through guano deposited by seabirds feeding in the Pacific Ocean affects the two dominant tree species of the coastal dry forests of northern Peru in contrasting ways. The non-nitrogen fixing species, *C. scabrida* may benefit from sea nutrient subsidies by incorporating guano-derived nitrogen into its foliar tissues, whereas *P. pallida*, capable of atmospheric fixation, does not.

## Introduction

The composition and dynamics of ecological communities in coastal marine and terrestrial ecosystems are partially driven by nutrient fluxes that link sea and adjacent land [1]–[5]. Some of the most striking examples of this interaction are found in the unproductive arid west coasts of North and South America where sea nutrient subsidies from the productive Pacific Ocean permeate through whole food webs affecting terrestrial plants [4], [6]–[8] and consumers [1], [7], [9]–[11], in various ways [12]–[14]. Marine nutrients reach land primarily through colonies of seabirds and marine mammals, or through organic debris, carcasses and algae deposited by tidal action [15], [16], and are further dispersed through trophic interactions [11], [14], [17], [18] and wind [4].

Seabird guano is a major nutrient path from the surrounding marine environments into terrestrial roosting and nesting sites worldwide [19]. It is well established that seabird guano increases soil nitrogen and phosphorus, although it can also affect soil pH, salinity and moisture [19]. These changes in soil characteristics generally translate into enhanced foliar nutrient levels and plant growth [20], especially when sufficient rainfall is available [6], [11], [12], [21]. However, at very high guano concentrations, plant cover can be reduced [21] or completely depleted [22], which has been attributed to ammonium toxicity [23] or acidic conditions [19]. Plant communities in seabird colonies are often dominated by cosmopolitan and ruderal species [19], [24] because the combined effects of seabirds include nutrient fertilization, physical disturbance and seed dispersal [19], [25]. Experimental manipulations of seabird guano have found contrasting responses across tree species, suggesting that differences in plant functional traits could help explain changes in plant composition in seabird-associated areas [26].

The overall effects of marine subsidies on arid coastal woody plant communities likely depend on how herbivores respond to plant nutrient enrichment and on how they are distributed along the sea-land gradient of nutrient fertilization. In pulsed systems, herbivores can limit plant growth [27], [28], but their effect on tree recruitment in arid coastal systems has not been directly assessed. Since herbivores usually respond positively to increasing plant nutrient content [29], we hypothesize that they can potentially offset the positive effects of sea nutrient subsides on tree seedling establishment.

In this paper, we assess the effects of seabird nutrient input and herbivory on tree seedling establishment in an arid coastal system. We hypothesised that non nitrogen-fixing tree species benefit more strongly from marine-derived nitrogen than nitrogen-fixing species since non-fixers depend on soil nitrogen sources and are not able to uptake atmospheric nitrogen through associations with rhizobial bacteria. We combined stable isotope analysis, field observations and experiments to test the following predictions: (1) marine nutrient fertilization has a positive effect on nitrogen assimilation of non-nitrogen fixing trees; (2) abundance of non-nitrogen fixing trees increases near the sea shore; (3) herbivory by lizards can offset the positive effects of nitrogen fertilization on tree seedling survival.

## Materials and Methods

### Study System

**Figure 1:**
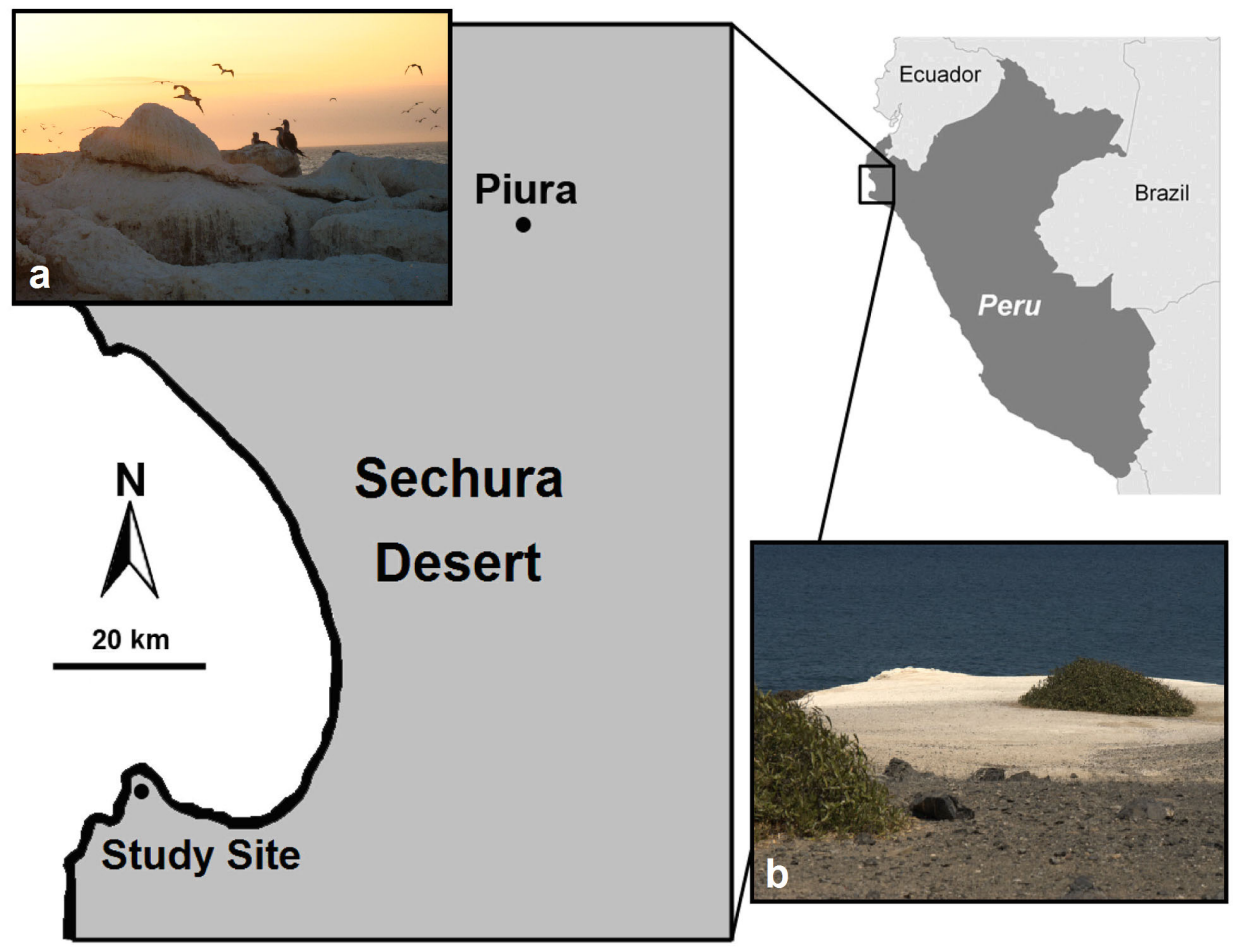
Location of the study site in Bayovar, north-western Peru. a) Guano accumulates in places where *Sula nebouxii* roost along shore (photograph G. Havik). b) *Capparis scabrida* growing in guano-rich soil (photograph A. Catenazzi).

We conducted our work in the coastal dry forests of north-western Peru, a region where the rich marine waters of the Peru-Chile current face the arid Sechura desert [1], [30]. Dry forests with an estimated surface of over 3 million ha expand through northern Peru and southern Ecuador [31]. These dry forests are dominated by *Prosopis pallida*, a nitrogen-fixing leguminous tree species, associated with other tree and shrub species such as the non-nitrogen fixing *Capparis scabrida* [32]. Vegetation becomes sparser closer to the coast (Fig. 1). We worked along the coast of Bayovar (5° 47′ S, 81° 04′ W; 8 m. a. s. l.; Fig. 1), near the oil pipeline terminal of PetroPerú and protected from human disturbance. This area is controlled by PetroPerú, and PetroPerú authorized our work in the area. We worked along the rocky seashore, rising about 7 m above the water and separated from the beach by cliffs. Large colonies of blue-footed boobies (*Sula nebouxii*) create abundant deposits of guano up to 50 cm thick (Fig. 1).

Rainfall is concentrated in the summer months (December-May) and is strongly influenced by El Niño Southern Oscillation (ENSO) events. Rainy ENSO events play a critical role in the regeneration of these forests, facilitating tree seedling establishment [27], [33], [34] and fast growth [35] that contributes to plants reaching a critical size at which they are less sensitive to herbivore damage [36]. Mean annual precipitation is ca. 50 mm (1961–1983; [37]), with high interannual variation (e.g. ranging from 2.8 mm in 1996 to 1639 mm in 1998). Mean annual temperature is 24.0°C, ranging between 21.1°C (August) and 27.9°C (February; [37]).

### Study Species

The two tree species are *Prosopis pallida* (Fabaceae) and *Capparis scabrida* (Capparaceae). *Prosopis pallida* is able to grow under arid conditions with at least 50 to 250 mm rainfall annually [32]. It can grow in nutrient-poor soils where its roots fix nitrogen in association with rhizobial bacteria [32], [38]. It is a fast growing tree with roots able to reach deep water sources up to 25 m below the surface [32]. Seed dispersal and germination are facilitated by a variety of animals [39]. Nevertheless, a high percentage of seeds remain dormant soon after falling from the tree [32]. Seedling establishment is greatly enhanced during rainy ENSO events [27], [35]. *Capparis scabrida* grows under a wide range of conditions, often in association with *P. pallida* [40]. Seeds are dispersed by foxes and birds and have high germination rates [40].

The most important herbivore in this region is *Dicrodon guttulatum* (Teiidae), a lizard endemic to north-western Peru that feeds preferentially on *P. pallida* [41] and strongly limits tree seedling establishment [27]. This is the most abundant herbivore at our site because livestock are not allowed within the PetroPerú concession.

### Soil Nutrients

We assessed soil nutrient concentrations along five transects perpendicular to the sea at increasing distances (10, 50, 100, 200 m) from the seashore. At each sampling location, we collected approximately 1 kg of soil from the top 30 cm of soil, and removed the rocks. Concentrations of NO_3_, NH_4_, PO_4_ and K were measured at the Resource Ecology Laboratory, University of Wageningen. Dried samples were extracted using a 0.01*M* CaCl_2_ solution previous to spectrophotometric determination of NO_3_ and NH_4_ by a Segmented Flow Analyzer (Skalar, Breda, The Netherlands). PO_4_ concentration was measured spectrophotometrically after digestion with H_2_O_2_. Concentrations of total nitrogen (N) and carbon (C) were measured in an Elemental Analyzer (EA 1108 CHN-O, Fisons Instruments) based on the dynamic combustion method. Potassium concentration was assessed using the ammonium acetate pH 7.0 protocol.

### Marine-derived Nitrogen in Plants and Soils

We measured nitrogen stable isotope ratios (δ^15^N) in soils and leaves of *P. pallida* and *C. scabrida* to assess the contribution of seabird-derived nitrogen in soil and foliar tissues. Values of δ^15^N have been used successfully to assess the contribution of seabird-derived nitrogen to coastal and insular food webs [6], [42], [43] because seabird guano typically has high δ^15^N values that can be over 30‰ [6], [44]. When terrestrial plants assimilate nitrogen from guano, their δ^15^N increases and approaches the δ^15^N of guano: for example in the Gulf of California, δ^15^N of the saltbush *Atriplex barclayana* was 5.26±0.85‰ on an island without seabirds, but 36.36±2.72‰ on a seabird island [6]. In the Sechura desert, δ^15^N values were −0.50±0.20‰ for *P. pallida* and 2.82±0.98‰ for *C. scabrida* in places that lack guano [33]. Thus, we expected foliar tissues of *P. pallida* and *C. scabrida* near seabird roosting sites to have δ^15^N values much higher than those measured in places lacking guano. For both tree species, we collected pooled samples of 5 leaves from 3 trees (at least 1.5 m tall, n = 15 leaves) growing at the same 4 distances from shore where other measurements were taken (10, 50, 100, 200 m) along six sea-inland transects (including the same transects used for sampling soil). We measured soil δ^15^N in the samples used for measurements of nutrient concentrations. All samples were oven dried at 55°C for 72 hours and pulverized in a sterilized mill prior to isotope analysis. Analysis of N concentration and δ^15^N was conducted on a Finnigan MAT Delta Plus continuous flow isotope ratio mass spectrometer at the Center for Stable Isotope Biogeochemistry, University of California, Berkeley. We analyzed 3–4 mg of leaves, and 10–75 mg of soil. Values of δ^15^N are expressed relative to its international standard, atmospheric air. We analyzed 18% of our samples in duplicate. The average standard deviation for replicate samples was 0.12‰.

### Spatial Tree Distribution

We evaluated the density of seedlings and trees of *P. pallida* and *C. scabrida* along the first 200 m from sea inland. We counted all seedlings and trees found in eight (300 m long × 60 m wide) transects perpendicular to shore. We divided each transect in six (50 m long × 60 m wide) plots.

### Seedling Establishment along a Natural Sea-inland Gradient of Soil Nitrogen Concentration

We designed a field experiment to assess the effects of distance from shore and herbivory pressure on early seedling survival of *P. pallida* and *C. scabrida*. At four distances from shore along a natural sea-inland gradient (10, 50, 100, 200 m), we planted 20 seedlings (10 of each tree species). Half of the seedlings (five of each species) were protected against herbivores. At each distance location, seedlings were planted at 25 cm distance from each other [35]. These treatments were replicated five times, along the same sea-inland transects where we measured soil nutrients and δ^15^N as described earlier. In summary, we planted 200 seedlings of each species for a total of 400 seedlings. Seedlings were protected against lizard browsing using plastic bottles (Ø: 10 cm, bottle top open). This method is commonly used in restoration projects in Peru because it is effective against herbivores [34] and experimental trials indicate that soil moisture content remains unaffected (File S1). Seedlings germinated from seeds collected locally and were transplanted when they were three weeks old in February 2009. We watered seedlings twice a week for the first seven weeks for a total of 270 mm, roughly equivalent to the rainfall during moderate ENSO events such as those in 1992 (235 mm, [34]) and 2002 (268 mm; [35]). No water was added during weeks with rainfall events of at least 5 mm. Total rainfall during the experimental time was 15 mm. Seedling survival was monitored regularly during three months.

### Statistical Analyses

We compared the soil nutrient concentrations (log-transformed) and the nitrogen stable isotope ratios (δ^15^N) in soils along the sea-inland nutrient gradient using general linear models with distance as a fixed factor and transect as a random factor. We tested for differences in leaf N concentrations and leaf δ^15^N for each tree species separately using general linear models including distance from shore as a fixed factor and transect as a random factor. We used generalized linear models to assess the effect of distance from shore on the number of naturally established plants for each species separately defining a Poisson distribution and using the factor distance from shore nested within transects.

To analyse the survival patterns of experimental seedlings, we used Cox proportional-hazards regression models, using the R-library survival [45]. We tested for differences in seedling survival for each tree species separately including distance from shore (log-transformed) and herbivory (protected or not) as covariables and cluster as a random factor to account for the grouping of 20 seedlings per location.

## Results

### Soil Nutrient Concentration along the Sea-inland Gradient

Soil nutrient concentrations were higher closer to shore (Table 1). Statistical tests indicate a significant effect of distance on [NO_3_] (F_3,12_ = 39.24; p<0.001), [NH_4_] (F_3,12_ = 6.60; p = 0.007), [PO_4_] (F_3,12_ = 7.84; p = 0.004), and on [K] (F_3,12_ = 18.23; p<0.001; see File S2 for details). Transects had no significant effects in any of the models.

**Table 1:** Nutrient concentrations in soil and leaves.

Nutrient Concentrations		Distance to shore							
		10 m		50 m		100 m		200 m	
**Soil**	Nitrate (µg/g)	94.49^a^	(40.56–887.75)	6.93^b^	(4.09–13.90)	1.84^c^	(1.44–2.04)	0.73^d^	(0.45–0.32)
	Ammonium (µg/g)	267.35^a^	(46.10–4993.15)	42.93^b^	(32.71–61.96)	66.61^ab^	(23.12–94.82)	50.64^ab^	(37.22–58.26)
	Phosphate (µg/g)	79.20^a^	(2.09–1114.80)	1.94^b^	(0.69–5.52)	1.30^b^	(0.30–3.47)	1.00^b^	(0.59–5.45)
	Potassium (µg/g)	425.00^a^	(233.00–1158.00)	95.60^b^	(58.40–98.00)	104.20^b^	(80.40–152.80)	93.00^b^	(76.20–152.80)
	%N	0.37^a^	(0.12–2.00)	0.12^ab^	(0.09–0.25)	0.12^b^	(0.10–0.18)	0.16^ab^	(0.12–0.26)
	%C	2.27^a^	(2.24–5.68)	1.45^ab^	(0.30–3.12)	1.03^b^	(0.49–1.75)	0.30^c^	(0.14–0.36)
**Leaves**	*C. scabrida* %N	1.80^a^	(1.58–2.25)	1.43^ab^	(1.27–2.05)	1.17^b^	(1.12–1.64)	1.43^ab^	(1.31–1.95)
	*C. scabrida* %C	41.73^a^	(40.90–42.76)	42.37^a^	(39.93–43.76)	41.18^a^	(39.55–42.57)	41.51^a^	(39.45–43.75)
	*P. pallida* %N	3.22^a^	(2.97–3.32)	3.23^a^	(2.91–3.91)	3.66^a^	(3.12–3.81)	3.48^a^	(3.13–3.97)
	*P. pallida* %C	44.58^a^	(42.59–45.73)	45.28^a^	(44.59–46.98)	46.09^a^	(45.35–47.28)	46.03^a^	(44.69–47.30)

Values are median and range (in parenthesis). Columns with same letters were not significantly different following post-hoc analysis (p>0.05).

### Nutrient Concentrations and δ^15^N of Soil and Tree Leaves

Leaves of *P. pallida* had higher N concentrations (3.41% ±0.31) than leaves of *C. scabrida* (1.50% ±0.30; Table 1). The two species differed in their leaf N concentration pattern along the sea shore-inland gradient. Whereas N concentrations of *P. pallida* did not change among sea-distance classes (F_3,11_ = 2.94; p = 0.08), leaves of *C. scabrida* contained more N in plants growing near shore (F_3,12_ = 15.75; p<0.001).

**Figure 2:**
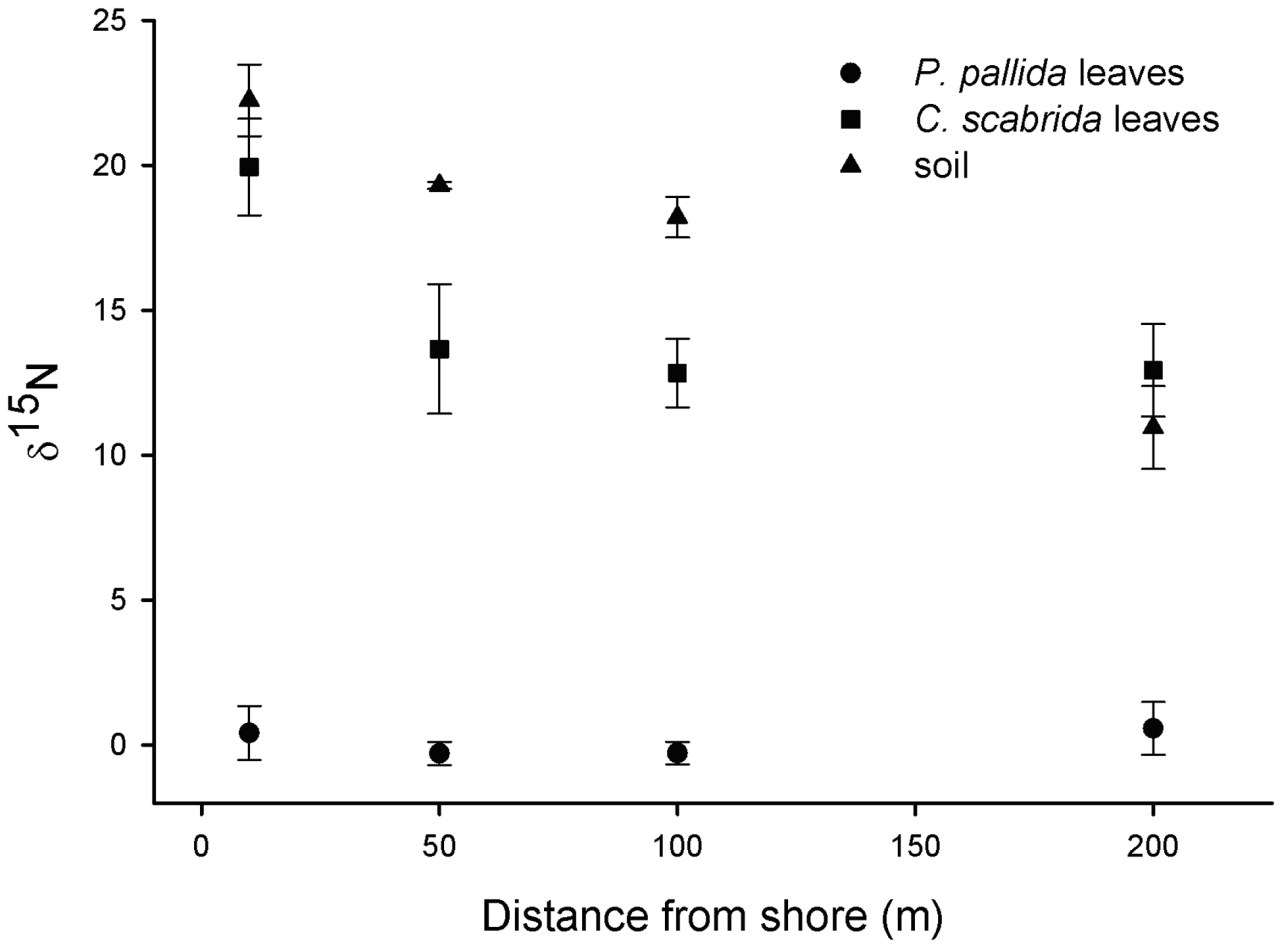
Nitrogen stable isotope ratios (δ^15^N; mean ± SE) for leaves of *Prosopis pallida* (circles), *Capparis scabrida* (squares), and soil samples (triangles) collected at 10, 50, 100 and 200 m from shore.

The δ^15^N of soil decreased with distance from shore (Fig. 2; F_3,10_ = 8.35; p = 0.004). Values of leaf δ^15^N for *C. scabrida* were more enriched than δ^15^N values for *P. pallida* (T- test = −14.71; p<0.001). Also the sea shore-inland pattern for leaf δ^15^N differed between the two species (Fig. 2). Whereas the δ^15^N for *P. pallida* did not vary among distance classes (Fig. 2; F_3,10_ = 0.67; p = 0.59), the δ^15^N for *C. scabrida* decreased with distance from shore (F_3,12_ = 3.54; p = 0.048). At a distance of 10 m from shore, soil was mostly composed of guano, and δ^15^N values averaged 22.24±2.75‰ for guano-rich soil, 19.94±1.66‰ for leaves of *C. scabrida*, and 0.87±0.82‰ for leaves of *P. pallida*.

### Spatial Distribution of Naturally Established Trees

**Figure 3:**
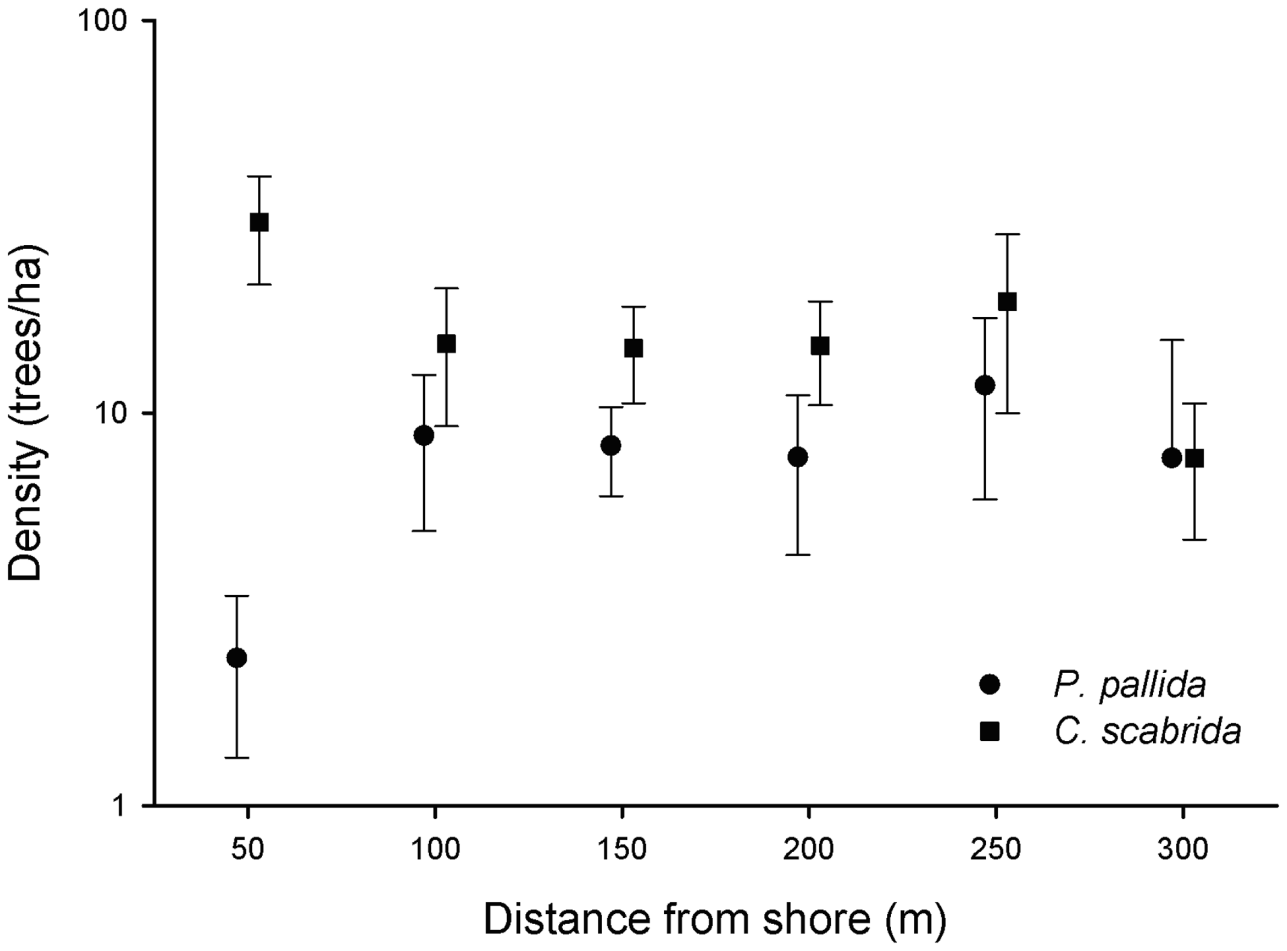
Density (mean ± SE; ha-1) of *Prosopis pallida* (circles) and *Capparis scabrida* (squares) seedlings and trees found along sea-inland transects.

Overall, the density of *C. scabrida* in our study plots was higher than the density of *P. pallida* (Fig. 3). We found differences in tree abundance among sea-distance classes for both *P. pallida* (Wald χ^2^ = 41.37; p = 0.028) and *C. scabrida* (Wald χ^2^ = 121.79; p<0.001). However the distribution pattern was different. Whereas the abundance of *C. scabrida* was higher close to shore, the abundance of *P. pallida* was lower. Within the first 50 m from shore, the density of *C. scabrida* was higher than the density of *P. pallida* (T-test; p = 0.01; Fig. 3).

### Survival of Planted Seedlings

**Figure 4:**
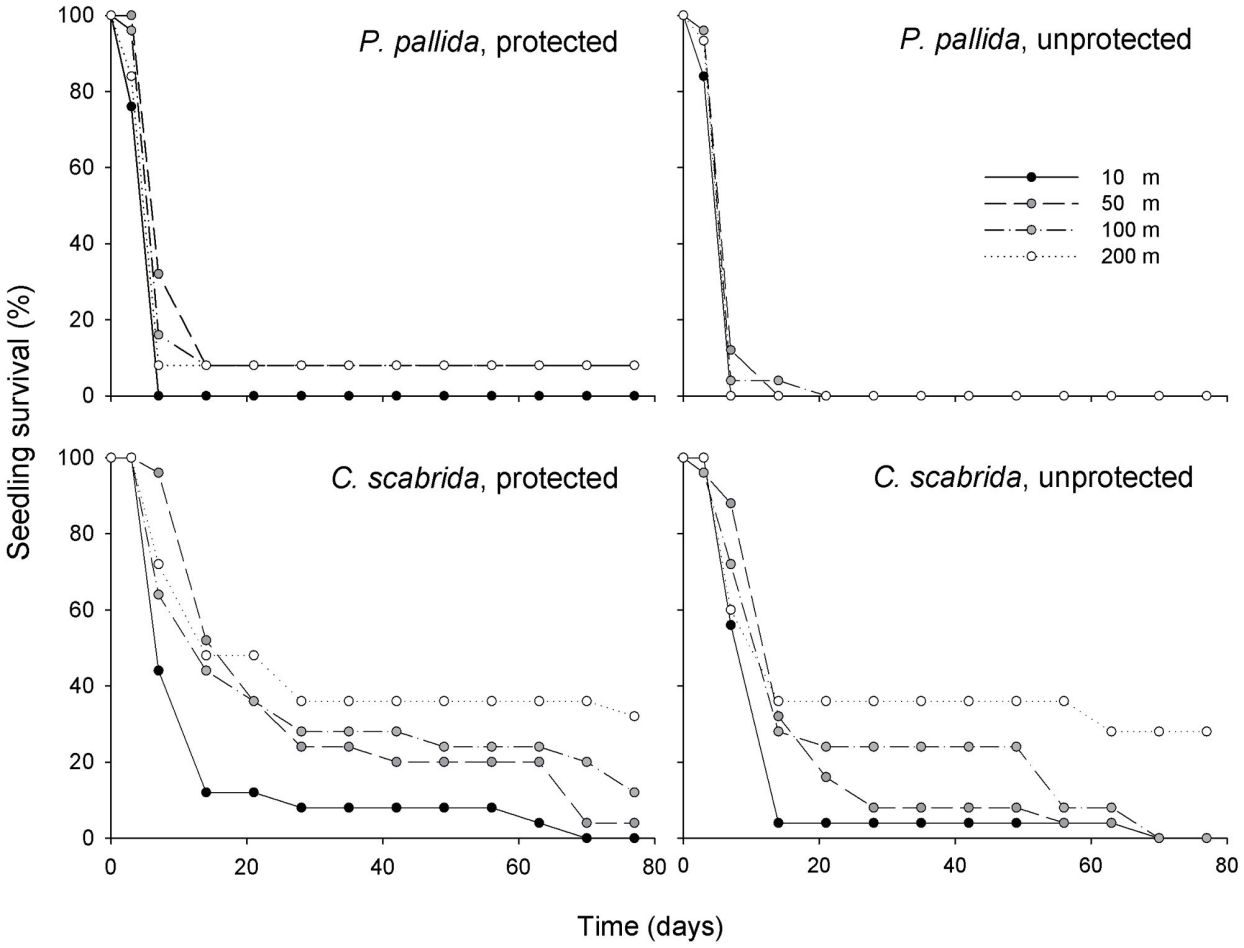
Survival (% individuals alive, n = 25) of seedlings of *Prosopis pallida* and *Capparis scabrida* in protected and unprotected experimental plots. Plots were located at increasing distance from shore (10, 50, 100 and 200 m). Overall survival (instead of mean and S.E.) illustrated here to facilitate comparison among distance classes.

The survival rate of *P. pallida* seedlings was lower than the survival of *C. scabrida* seedlings; Fig. 4). Protection from lizards had no significant effect on the survival of the two species (Robust-test; p = 0.085 and p = 0.106 for *P. pallida* and *C. scabrida* respectively). Since the effects of herbivores were not significant, we modelled seedling survival as a function of species and distance from shore including a species *distance interaction. We found that only the interaction was significant (p<0.05) indicating that the species responded differently to the sea distance gradient which is also reflected in the models for each species separately. Distance from shore had a positive significant effect on the survival rate of *C. scabrida* (Robust-test; p = 0.0004; Fig. 4) but no effect on the survival of *P. pallida* seedlings (Robust-test; p = 0.826; Fig. 4).

## Discussion

Our study did not find a significant effect of herbivory on tree seedling survival in a tropical dry forest subsidized by seabird guano. Woody plant species are common in tropical ecosystems receiving large amounts of seabird guano [26], [43], [46]. Previous studies have documented high densities of lizards in systems subsidized by marine-derived resources [9], [13], [47]. Therefore, we anticipated a positive effect of seabird-derived subsidies on the strength of top-down interactions at our study site. Our negative results confirm that it is difficult to predict the effect of seabird-derived resources on species interactions, or to generalize their effects on food web composition and functioning [48].

Our focal lizard species *Dicrodon guttulatum* is a widespread lizard in the Sechura desert [41] and has been identified as a major limiting factor for the regeneration of inland dry forests in northern Peru [27], [34]. At our coastal site experimental protection from lizard herbivory did not affect survival of the two tree species. Therefore, top-down control by herbivorous lizards seems to be weaker in coastal dry forests than it is in inland dry forests. This difference might be explained by lower lizard density in coastal sites (unpublished data). *Dicrodon guttulatum* feeds primarily on *P. pallida* [41], and the abundance of *P. pallida* can be much higher at inland sites than it is at our coastal site, presumably supporting larger populations of lizards.

Although herbivory did not play a role in seedling survival, we observed differences in the distribution of the two tree species at our coastal site. Nutrient and stable isotopic analyses suggest alternative explanations for the observed pattern of increased dominance of the non-fixing species close to shore. The observed patterns of δ^15^N and N concentrations in foliar tissue (Fig. 2) suggest a positive response to increased soil N concentrations near shore for the non-fixing *C. scabrida* but not for the fixing *P. pallida*. This result is in line with our hypothesis that sea-nutrient fertilization has a positive effect on N assimilation of the non-N fixer. The higher concentrations of NH_4_ and NO_3_ close to shore indicate that more soil N is available to plants near shore. Leaf tissues of *C. scabrida* trees growing near shore had elevated δ^15^N values, suggesting assimilation of marine-derived N because δ^15^N in guano-rich soil had similarly high values (Fig. 2). Higher N concentrations in *C. scabrida* tissue near shore support this result. In this coastal ecosystem, *C. scabrida* is the most abundant plant species suggesting that it can be an important vector of marine-derived nutrients to the terrestrial trophic food web through diverse types of consumers feeding on its leaves or fruits (e.g. foxes, goats, birds and insects).

The δ^15^N of *P. pallida* was always close to zero, suggesting that atmospheric N was the source. Moreover, the N concentrations in foliar tissue varied little between *P. pallida* plants growing in N-rich soils near shore and plants growing further inland where N concentrations were lower. These results are particularly interesting compared to previous studies. Elevated soil N concentrations could inhibit N fixation [50], for example *Prosopis juliflora* had lower N-fixation rates and higher δ^15^N under saline conditions [49]. Our data suggest that *P. pallida* assimilates atmospheric-fixed N regardless of soil N concentration in the soil and distance from shore. An alternative hypothesis, differences in fractionation during N assimilation, is unlikely to explain these patterns because our aggregated samples pooled leaves from different parts of the plants and from several individuals at each distance class, and because we collected replicated samples along a linear gradient of guano concentration. Therefore, given the lack of correspondence between the spatial patterns of δ^15^N in soil and stability of δ^15^N in leaves of *P. pallida*, the most parsimonious explanation is that *P. pallida* does not assimilate N from the soil even in places where NH_4_ and NO_3_ concentrations are high.

The density of naturally established trees of both species suggests a response to the sea-inland nutrient patterns consistent with results from the stable isotope analysis. Near shore, the density of *C. scabrida* was higher than the density of *P. pallida*. Density of *P. pallida* was low near shore, increased between 50 and 100 m, and remained relatively constant and similar to *C. scabrida* further away. These data suggest that *C. scabrida* growing near shore benefits more from high nutrient concentrations than *P. pallida*. High nutrient concentration may affect the competitive ability of the two tree species by promoting growth or survival of the non-fixing species. Our seedling transplant experiment did not detect an effect of distance from shore on survival. Therefore, high nutrient concentrations are likely to enhance growth rate of the non-fixer without affecting the growth rate of the fixer, as suggested by patterns of increased tissue N concentrations in *C. scabrida* near shore, and no change in tissue N concentration in *P. pallida*. The positive effects of marine nutrient subsidies on *C. scabrida* were restricted to areas very close to the sea guano deposits. Remarkably localized effects have also been found in other seabird studies worldwide [51], as well as in the transfer of marine-derived nitrogen accumulated and transported by salmon to riverine forests of North America [52], [53].

An alternative hypothesis to explain increased dominance of the non-fixer near shore is that the fixer is suppressed by the guano nitrogen due to toxicity. This hypothesis relies on the assumption that *P. pallida* are getting their nitrogen from fixation rather than from the guano-rich soil near shore, as opposed to *C. scabrida* incorporating nitrogen from guano-rich soil. This assumption is supported by constant δ^15^N values in the fixer and decreasing δ^15^N in the non-fixer with distance from shore. Moreover, in a small laboratory experiment on the germination of *P. pallida* (n = 50 seeds), we found that seeds germinated better in soils coming from ≥50 m from shore than in guano-rich soils collected near shore [54]. However, our field experiment results show that seedling establishment close to shore is difficult for both tree species. The planted seedlings of *C. scabrida* did not grow well and ultimately died near shore, whereas the survival of *P. pallida* was very low throughout the 200 m sea-inland gradient (Fig. 4).

The toxic effect of N for both species’ seedlings could be explained either by the acidifying effect of ammonia on the soil [55], by N toxicity, or by a combination of both [19]. The effects of P on nitrogen and non-nitrogen fixers are complex. Nitrogen fixing trees require relatively high supplies of P for the process of fixation [56]. In natural populations, the rates of N fixation increase with increasing soil P availability [57]. However, under non-limiting N conditions, experimental evidence shows that N acquisition strategies change with P supply, and fixers rely less on N fixation [58].

It has been suggested that the toxicity effects of guano may be more common in hyperarid regions [19]. We speculate that nutrient leaching during rainy years could drop nutrient concentrations below toxic levels. This would explain the apparent discrepancy between the natural distribution patterns and the outcome of the field experiment in our study system. Indeed the benefits of marine nutrient fertilization on arid plant communities can be particularly strong during rainy years, like those associated to ENSO, when water availability is not limiting [11], [19]. It has been suggested that this positive effect can build up organic matter that in turn improves soil water retention and, as a consequence, long-term guano accumulation can have a positive effect on plant productivity even in dry years [8]. However, the relationship between guano accumulation and soil moisture is not always positive [19].

Salinity is another environmental factor that could change along the sea-inland gradient. Earlier work indicates that both *P. pallida* [32] and *C. scabrida* [40] can tolerate high soil salinity, but future manipulative experiments may contribute to further separate the role of nutrients and salinity.

We conclude that the input of marine-derived N through guano deposited by seabirds feeding in the Pacific Ocean affects the two dominant tree species of the coastal dry forests of northern Peru in contrasting ways. The non-N fixing species, *C. scabrida* may benefit from sea nutrient subsidies by incorporating marine-derived N into its foliar tissues, whereas *P. pallida*, capable of atmospheric N fixation, does not.

## Supporting Information

File S1 **Assessment of microclimate changes introduced by the herbivore exclusions.**
(DOCX)

File S2 **Results of general linear models using distance to shore as fixed factor and transect as a random factor.**
(DOCX)
