# Supplementary material for: Seabird Nutrient Subsidies Benefit Non-Nitrogen Fixing Trees and Alter Species Composition in South American Coastal Dry Forests
Source: PLoS One. 2014 Jan 22;9(1):e86381. doi: 10.1371/journal.pone.0086381 (PMC3899251; doi:10.1371/journal.pone.0086381)
Supplement: File S1 — Assessment of microclimate changes introduced by the herbivore exclusions. (DOCX) [file pone.0086381.s001.docx]

**Supporting information**

**File S1.** Assessment of microclimate changes introduced by the herbivore exclusions

We conducted a small experiment to test if the bottles used to exclude herbivores change the microclimatic conditions. We designed a factorial experiment with two treatments: bottle and open replicated 10 times. We tested for changes in soil temperature, air temperature and soil water content.

*Methods*

This experiment was conducted between Monday May 28^th^ and Monday June 4^th^ 2012. We marked 10 locations in an open area within the experimental site of the UDEP (north Peru). In each location we marked two points: one with a bottle as the one used to exclude herbivores in the field experiment (main text), and one without the bottle (Fig. 1). Bottles were buried 3 to 5 cm deep and separated at least by 1 meter.

To compare the microclimatic conditions inside and outside the bottles, we measured air and soil temperature daily at 10:00 am for 8 days in each of the 20 experimental points (10 pairs, bottle-no bottle). We also registered soil temperature continuously in 2 pairs (bottle-no bottle) at 3-5 cm below the surface. Water content was estimated gravimetrically taking soil samples at 5 cm deep using standard methods (we weighted them, dried them in a stove at 100°C for 72 hours, and then weighted them again to assess how much water had been evaporated).


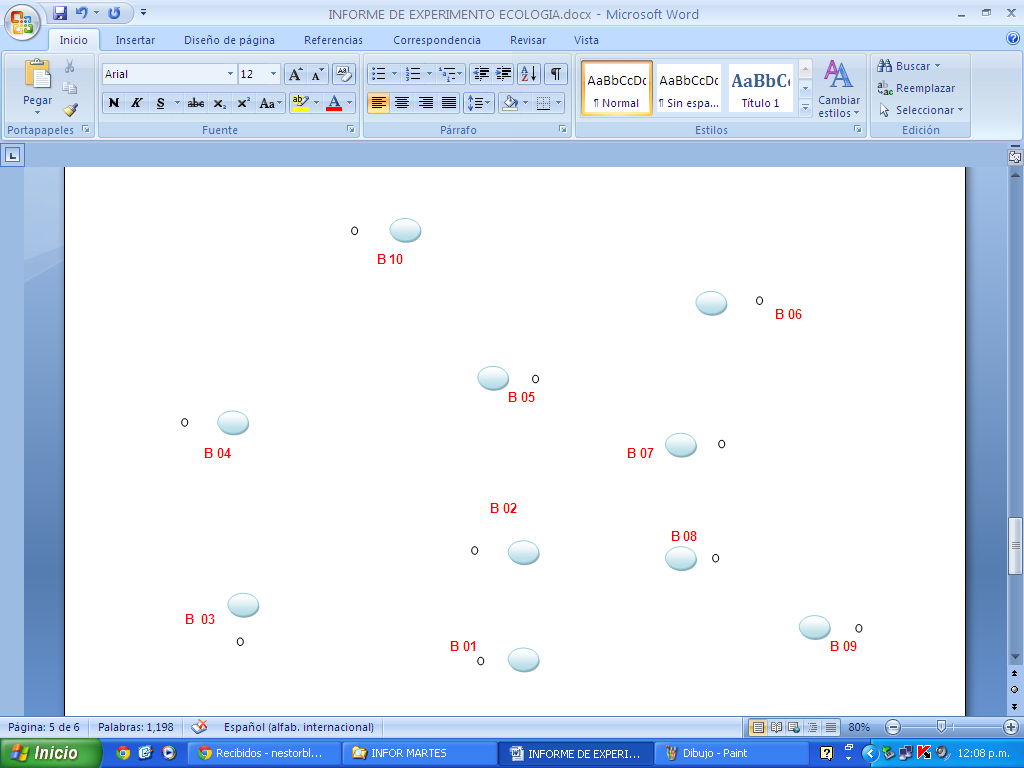


Locations of the 10 experimental pairs: bottle (large light blue circle)-no bottle (small circle).

*Results*

We compared the inside and outside temperatures with a paired samples T-test. Air and soil temperatures were in average 1.3°C higher inside the bottles (paired samples T-test: p < 0.001 for both air and soil temperature). We tested for changes in soil water content by comparing the soil weight before and after the 8 experimental days inside and outside the bottles. We did not find a difference in soil moisture pre and post experiment (Paired samples T-test: p = 0.251).

**File S2.** Results of general linear models using distance to shore as fixed factor and transect as a random factor.

**1. Soil Contents**

**1.1 NO_3_ (log-transformed)**

| **Tests of Between-Subjects Effects** | | | | | | |
| --- | --- | --- | --- | --- | --- | --- |
| Dependent Variable:LogNO3 | | | | | | |
| Source | | Type III Sum of Squares | df | Mean Square | F | Sig. |
| Intercept | Hypothesis | 11.898 | 1 | 11.898 | 236.424 | .000 |
|  | Error | .201 | 4 | .050^a^ |  |  |
| Distance | Hypothesis | 15.434 | 3 | 5.145 | 39.235 | .000 |
|  | Error | 1.574 | 12 | .131^b^ |  |  |
| Transect | Hypothesis | .201 | 4 | .050 | .384 | .816 |
|  | Error | 1.574 | 12 | .131^b^ |  |  |
| Distance * Transect | Hypothesis | 1.574 | 12 | .131 | . | . |
|  | Error | .000 | 0 | .^c^ |  |  |
| a. MS(Transect)  b. MS(Distance * Transect)  c. MS(Error) | | | | | | |

**1.2 NH_4_ (log-transformed)**

| **Tests of Between-Subjects Effects** | | | | | | |
| --- | --- | --- | --- | --- | --- | --- |
| Dependent Variable: logNH4 | | | | | | |
| Source | | Type III Sum of Squares | df | Mean Square | F | Sig. |
| Intercept | Hypothesis | 73,566 | 1 | 73,566 | 434,173 | ,000 |
|  | Error | ,678 | 4 | ,169^a^ |  |  |
| Distance | Hypothesis | 3,109 | 3 | 1,036 | 6,596 | ,007 |
|  | Error | 1,886 | 12 | ,157^b^ |  |  |
| Transect | Hypothesis | ,678 | 4 | ,169 | 1,078 | ,410 |
|  | Error | 1,886 | 12 | ,157^b^ |  |  |
| Distance * Transect | Hypothesis | 1,886 | 12 | ,157 | . | . |
|  | Error | ,000 | 0 | .^c^ |  |  |
| a. MS(Transect) | | | | | | |
| b. MS(Distance * Transect) | | | | | | |
| c. MS(Error) | | | | | | |

**1.3 PO_4_ (log-transformed)**

| **Tests of Between-Subjects Effects** | | | | | | |
| --- | --- | --- | --- | --- | --- | --- |
| Dependent Variable:LogPO4 | | | | | | |
| Source | | Type III Sum of Squares | df | Mean Square | F | Sig. |
| Intercept | Hypothesis | 5.251 | 1 | 5.251 | 8.772 | .041 |
|  | Error | 2.394 | 4 | .599^a^ |  |  |
| Distance | Hypothesis | 8.059 | 3 | 2.686 | 7.835 | .004 |
|  | Error | 4.114 | 12 | .343^b^ |  |  |
| Transect | Hypothesis | 2.394 | 4 | .599 | 1.746 | .205 |
|  | Error | 4.114 | 12 | .343^b^ |  |  |
| Distance * Transect | Hypothesis | 4.114 | 12 | .343 | . | . |
|  | Error | .000 | 0 | .^c^ |  |  |
| a. MS(Transect)  b. MS(Distance * Transect)  c. MS(Error) | | | | | | |

**1.4 K (log-transformed)**

| **Tests of Between-Subjects Effects** | | | | | | |
| --- | --- | --- | --- | --- | --- | --- |
| Dependent Variable:LogK | | | | | | |
| Source | | Type III Sum of Squares | df | Mean Square | F | Sig. |
| Intercept | Hypothesis | 94.075 | 1 | 94.075 | 9885.063 | .000 |
|  | Error | .038 | 4 | .010^a^ |  |  |
| Distance | Hypothesis | 2.136 | 3 | .712 | 18.225 | .000 |
|  | Error | .469 | 12 | .039^b^ |  |  |
| Transect | Hypothesis | .038 | 4 | .010 | .244 | .908 |
|  | Error | .469 | 12 | .039^b^ |  |  |
| Distance * Transect | Hypothesis | .469 | 12 | .039 | . | . |
|  | Error | .000 | 0 | .^c^ |  |  |
| a. MS(Transect)  b. MS(Distance * Transect)  c. MS(Error) | | | | | | |

**1.5 Soil δ^15^N**

| **Tests of Between-Subjects Effects** | | | | | | |
| --- | --- | --- | --- | --- | --- | --- |
| Dependent Variable:ISOsoil | | | | | | |
| Source | | Type III Sum of Squares | df | Mean Square | F | Sig. |
| Intercept | Hypothesis | 4990.944 | 1 | 4990.944 | 871.608 | .000 |
|  | Error | 24.733 | 4.319 | 5.726^a^ |  |  |
| Distance | Hypothesis | 326.856 | 3 | 108.952 | 8.351 | .004 |
|  | Error | 130.467 | 10 | 13.047^b^ |  |  |
| Transect | Hypothesis | 22.408 | 4 | 5.602 | .429 | .784 |
|  | Error | 130.467 | 10 | 13.047^b^ |  |  |
| Distance * Transect | Hypothesis | 130.467 | 10 | 13.047 | . | . |
|  | Error | .000 | 0 | .^c^ |  |  |
| a. .983 MS(Transect) + .017 MS(Distance * Transect)  b. MS(Distance * Transect)  c. MS(Error) | | | | | | |

**2. Leaf contents**

**2.1 *P. pallida* N content**

| **Tests of Between-Subjects Effects^d^** | | | | | | |
| --- | --- | --- | --- | --- | --- | --- |
| Dependent Variable:NPercent | | | | | | |
| Source | | Type III Sum of Squares | df | Mean Square | F | Sig. |
| Intercept | Hypothesis | 208.130 | 1 | 208.130 | 1328.366 | .000 |
|  | Error | .806 | 5.145 | .157^a^ |  |  |
| Distance | Hypothesis | .548 | 3 | .183 | 2.940 | .080 |
|  | Error | .684 | 11 | .062^b^ |  |  |
| Transect | Hypothesis | .801 | 5 | .160 | 2.576 | .089 |
|  | Error | .684 | 11 | .062^b^ |  |  |
| Distance * Transect | Hypothesis | .684 | 11 | .062 | . | . |
|  | Error | .000 | 0 | .^c^ |  |  |
| a. .964 MS(Transect) + .036 MS(Distance * Transect)  b. MS(Distance * Transect)  c. MS(Error)  d. Species = P. pallida | | | | | | |

**2.2 *C. scabrida* N content**

| **Tests of Between-Subjects Effects^d^** | | | | | | |
| --- | --- | --- | --- | --- | --- | --- |
| Dependent Variable:NPercent | | | | | | |
| Source | | Type III Sum of Squares | df | Mean Square | F | Sig. |
| Intercept | Hypothesis | 49.326 | 1 | 49.326 | 270.003 | .000 |
|  | Error | .918 | 5.024 | .183^a^ |  |  |
| Distance | Hypothesis | .812 | 3 | .271 | 15.758 | .000 |
|  | Error | .206 | 12 | .017^b^ |  |  |
| Transect | Hypothesis | .935 | 5 | .187 | 10.890 | .000 |
|  | Error | .206 | 12 | .017^b^ |  |  |
| Distance * Transect | Hypothesis | .206 | 12 | .017 | . | . |
|  | Error | .000 | 0 | .^c^ |  |  |
| a. .975 MS(Transect) + .025 MS(Distance * Transect)  b. MS(Distance * Transect)  c. MS(Error)  d. Species = C. scabrida | | | | | | |

**2.3 *P. pallida* δ^15^N content**

| **Tests of Between-Subjects Effects^d^** | | | | | | |
| --- | --- | --- | --- | --- | --- | --- |
| Dependent Variable:DeltaN | | | | | | |
| Source | | Type III Sum of Squares | df | Mean Square | F | Sig. |
| Intercept | Hypothesis | 1.374 | 1 | 1.374 | .551 | .489 |
|  | Error | 13.363 | 5.361 | 2.493^a^ |  |  |
| Distance | Hypothesis | 3.592 | 3 | 1.197 | .668 | .590 |
|  | Error | 17.912 | 10 | 1.791^b^ |  |  |
| Transect | Hypothesis | 12.641 | 5 | 2.528 | 1.411 | .300 |
|  | Error | 17.912 | 10 | 1.791^b^ |  |  |
| Distance * Transect | Hypothesis | 17.912 | 10 | 1.791 | . | . |
|  | Error | .000 | 0 | .^c^ |  |  |
| a. .952 MS(Transect) + .048 MS(Distance * Transect)  b. MS(Distance * Transect)  c. MS(Error)  d. Species = P. pallida | | | | | | |

**2.4 *C. scabrida* δ^15^N content**

| **Tests of Between-Subjects Effects^d^** | | | | | | |
| --- | --- | --- | --- | --- | --- | --- |
| Dependent Variable:DeltaN | | | | | | |
| Source | | Type III Sum of Squares | df | Mean Square | F | Sig. |
| Intercept | Hypothesis | 4130.056 | 1 | 4130.056 | 214.371 | .000 |
|  | Error | 98.685 | 5.122 | 19.266^a^ |  |  |
| Distance | Hypothesis | 96.791 | 3 | 32.264 | 3.540 | .048 |
|  | Error | 109.371 | 12 | 9.114^b^ |  |  |
| Transect | Hypothesis | 97.655 | 5 | 19.531 | 2.143 | .130 |
|  | Error | 109.371 | 12 | 9.114^b^ |  |  |
| Distance * Transect | Hypothesis | 109.371 | 12 | 9.114 | . | . |
|  | Error | .000 | 0 | .^c^ |  |  |
| a. .975 MS(Transect) + .025 MS(Distance * Transect)  b. MS(Distance * Transect)  c. MS(Error)  d. Species = C. scabrida | | | | | | |
